# Exponential Relationship Between Maximal Apnea Duration and Exercise Intensity in Non-apnea Trained Individuals

**DOI:** 10.3389/fphys.2021.815824

**Published:** 2022-01-25

**Authors:** Alexandre Guimard, Fabrice Joulia, Fabrice Prieur, Gauthier Poszalczyk, Kader Helme, François J. Lhuissier

**Affiliations:** ^1^Université Sorbonne Paris Nord, Hypoxie et Poumon, H&P, INSERM, UMR 1272, Bobigny, France; ^2^Département STAPS, Université Sorbonne Paris Nord, Bobigny, France; ^3^Center for Cardiovascular and Nutrition Research (C2VN), INSERM 1263, INRAE 1260, Aix Marseille Université, Marseille, France; ^4^UFR STAPS, Toulon, France; ^5^Université Paris-Saclay, CIAMS, Orsay, France; ^6^Université d’Orléans, CIAMS, Orléans, France; ^7^Assistance Publique – Hôpitaux de Paris, Hôpitaux Universitaires Paris Seine-Saint-Denis, Hôpital Jean Verdier, Médecine de l’Exercice et du Sport, Bondy, France

**Keywords:** aerobic training, dynamic apnea, oxygen uptake, exponential function, oxygen saturation, lactatemia, RPE, subjective feeling

## Abstract

It is well known that the duration of apnea is longer in static than in dynamic conditions, but the impact of exercise intensity on the apnea duration needs to be investigated. The aim of this study was to determine the relationship between apnea duration and exercise intensity, and the associated metabolic parameters. Ten healthy active young non-apnea trained (NAT) men participated in this study. During the first visit, they carried out a maximum static apnea (SA) and a maximal progressive cycle exercise to evaluate the power output achieved at peak oxygen uptake (PVO_2_peak). During the second visit, they performed four randomized dynamic apneas (DAs) at 20, 30, 40, and 50% of PVO_2_peak (P20, P30, P40, and P50) preceded by 4 min of exercise without apnea. Duration of apnea, heart rate (HR), arterial oxygen saturation (SpO_2_), blood lactate concentration [La], rating of perceived exertion (RPE), and subjective feeling were recorded. Apnea duration was significantly higher during SA (68.1 ± 23.6 s) compared with DA. Apnea duration at P20 (35.6 ± 11.7 s) was higher compared with P30 (25.6 ± 6.3 s), P40 (19.2 ± 6.7 s), and P50 (16.9 ± 2.5 s). The relationship between apnea duration and exercise intensity followed an exponential function (*y* = 56.388e^–0.025^*^x^*). SA as DA performed at P20 and P30 induces a bradycardia. Apnea induces an SpO_2_ decrease which is higher during DA (−10%) compared with SA (−4.4%). The decreases of SPO_2_ recorded during DA do not differ despite the increase in exercise intensity. An increase of [La] was observed in P30 and P40 conditions. RPE and subjective feeling remained unchanged whatever the apnea conditions might be. These results suggest that the DA performed at 30% of VO_2_peak could be the best compromise between apnea duration and exercise intensity. Then, DA training at low intensity could be added to aerobic training since, despite the moderate hypoxia, it is sufficient to induce and increase [La] generally observed during high-intensity training.

## Introduction

Hypoxia is commonly used as a training modality in many sports. To avoid going to altitude or using expensive devices simulating a hypoxic environment, other alternatives have emerged such as training with voluntary hypoventilation (at low pulmonary volumes) (Woorons et al., 2007, 2008, 2010) or even apnea. Indeed, apnea certainly exists as a sport, but it also appears as a new modality of training in water sports but also on land. Apnea is then considered as a model of decreased O_2_ availability that can spontaneously be compared with hypoxia as a model of hypoxic stimulus. While apnea training could be an effective alternative to hypobaric or normobaric hypoxia to increase aerobic and/or anaerobic performance (Lemaître et al., 2010), this type of training is most often used empirically. Apnea causes a well-known cardiovascular adaptation called the “diving reflex” (Lindholm and Lundgren, 2009). In humans, the diving response includes bradycardia, peripheral vasoconstriction, increased arterial blood pressure, reduced cardiac output and blood flow, and increased sympathetic activity triggered in response to cessation of ventilation (Sterba and Lundgren, 1988; Gooden, 1994; Foster and Sheel, 2005; Lindholm and Lundgren, 2009). An active contraction of the spleen was also considered as part of this diving response (Hurford et al., 1990). The diving response, which would aim to save O_2_, causes a distribution of pulmonary and blood O_2_ stocks preferentially toward the heart and the brain (Lindholm and Lundgren, 2009) and can, therefore, be considered as an important mechanism of defense against damage from hypoxia (Alboni et al., 2011).

During physical exercise, the energy is mainly produced through the use of O_2_, and many studies have shown that the main factor involved in physical performance is the ability of the body to bring oxygen to the muscles and also to the brain. For equivalent apnea durations, there is a greater decrease in arterial oxygen saturation (SpO_2_) (Delahoche et al., 2005) during dynamic apnea (DA) (Ahn et al., 1989; Lindholm et al., 1999, 2002; Joulia et al., 2009; Breskovic et al., 2011; Guimard et al., 2014, 2017, 2018) than during static apnea (SA) (Palada et al., 2007; Andersson et al., 2008; Kiviniemi et al., 2012; Costalat et al., 2013; Engan et al., 2013). This drop is greater in free divers due to the longer duration of the apnea (Palada et al., 2007).

More broadly, if apnea alone (i.e., in the air) is sufficient to trigger the diving response, the observed response is modulated according to various factors such as immersion, water temperature, hypoxia, or even training (Stromme et al., 1970; Schuitema and Holm, 1988; Lindholm and Lundgren, 2009). It is important to note that the longest apneas and the most pronounced cardiovascular adjustments were observed in trained participants (Andersson and Schagatay, 1998), suggesting a link between the duration of the apnea and the importance of the diving reflex (Caspers et al., 2011).

For a training purpose, the coaches empirically carry out exercises of varying intensity and duration of apnea, i.e., either exercises at very high intensity therefore of shorter duration of exercise and apnea or exercises at lower intensity therefore of longer duration of exercise and apnea. It is well known that the duration of apnea is longer in static than in dynamic conditions, but the impact of exercise intensity on the apnea duration needs to be investigated. This interaction is still poorly understood because very less was studied. Wein’s team studied the physiological effects of dynamics apneas as a discipline in recreational or competitive breath-hold diving (Wein et al., 2007). For this, participants trained in apnea performed maximum apnea with their face submerged at rest and simultaneously with exercises of different intensities (40, 80, and 120 W) on an ergocycle. The results seem to indicate that the duration of apnea decreased with the exercise power according to an exponential function (data not mentioned) of the maximal apnea duration. The precise knowledge of this interaction could be used as a basis for the development of apnea training, because it is likely to increase induced cellular hypoxia and therefore the adaptive responses to training. In addition, it might be interesting to test this interaction on untrained participants in apnea and in the air for wider application in the field of training. Thus, the aim of this study was to determine the relationship between apnea duration and exercise intensity, and the apnea exercise metabolic effects. We hypothesized that there is an optimal exercise intensity during DA to induce metabolic effect and an exponential relationship between exercise intensity and duration of apnea.

## Materials and Methods

### Participants

The experimental group consisted of 10 healthy male students: 21 ± 3.3 years (weight: 69.3 ± 5.9 kg and height 176.4 ± 5.3 cm). None of them had been trained in apnea. However, participants were not naïve to apnea and well-trained in physical activities. All participants were students at the Faculty of Sport Sciences. During their studies, it was mandatory to practice swimming and water rescue that are activities known to require apnea phases. Furthermore, during the recruitment, all the volunteers were asked if they feel comfortable with apnea. Only the one comfortable participated in this study. Finally, the participants who consent to participate indicated that they were able to maintain at least 1 min of SA. All of them were non-smokers, and none of them were taking any medication or had a family history of cardiac, respiratory, or metabolic pathology. After being informed of the nature of the experiments, all the participants gave their informed consent to participate in the protocol, and all procedures were designed according to the declaration of Helsinki and approved by the Committee for the Protection of Persons Tours—Région Centre—Ouest 1.

### Procedures

Participants performed, after a day off from intense exercise, different tests on two visits separated by 48 h. The participants consumed no caffeine or alcohol during the preceding 24 h of the experiment.

During the first visit, the participants were informed about the experiment. After the medical consultation that included anthropometric measurements and basal data of cardiovascular parameters such as heart rate (HR), each subject performed on an ergocycle (Ergoline 200) a maximum SA preceded by 5 min of rest, followed by an incremental exercise test. The latter consisted of 5 min of rest sitting on the same cycle ergometer followed by 3 min warm up at 60 W and by an intensity increase of 30 W every 2 min (60 rpm) until exhaustion to determine the peak oxygen uptake (VO_2_peak) and the power output achieved at VO_2_peak (PVO_2_peak). This maximal exercise test was followed by 6 min of passive sitting recovery. These two tests were separated by 15 min of passive recovery.

**FIGURE 1:**
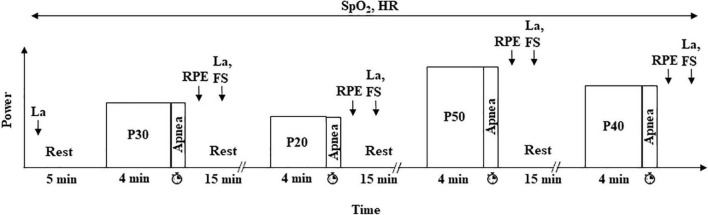
Experimental protocol (visit 2). Participants performed four randomized exercises at 20, 30, 40, and 50% of PVO_2_peak (P20, P30, P40, and P50, respectively) followed by a maximal dynamic apnea at the same power. HR and SpO_2_ were continuously monitored, and [La] was measured at rest and 2 min after the end of each dynamic apnea. RPE and FS were estimated 1 and 2 min after exercise, respectively. The duration of each apnea was measured.

During the second visit (Figure 1), participants started with 5 min of rest. Then, they performed four blocks of exercise at 60 rpm and at a power corresponding to 20, 30, 40, and 50% of PVO_2_peak (P20, P30, P40, and P50, respectively). Each block consisted of 4 min of exercise without apnea at a specific power to reach a steady state followed by a maximum DA (i.e., the longest time that a participant could endure in apnea at that power). Each block was separated from the following one by 15 min of passive recovery. The order of the blocks was randomized. All the apneas were performed after a maximal inspiration.

### Measurements

Gas exchanges were continuously measured for the analysis during the incremental cycle exercise by a breath-by-breath gas exchange measurement system (Jaeger; CareFusion, Germany). Each apnea duration was measured using a manual stopwatch. HR was continuously monitored and recorded at rest, during exercise, and during the recovery period (PhysioFlow^®^, Manatec type PF05L1, Paris, France). In addition, the arterial O_2_ saturation (SpO_2_) was continuously evaluated using a pulse oximeter with an ear sensor of the same device at rest for each apnea condition, during SA and DA, and during the post-exercise rest period in visit 2. The minimal value of SpO_2_ was determined (SpO_2_min) during SA and DA and during the 30 s after the end of each apnea condition. For the measurement of lactatemia ([La]) (ABL 700 Radiometer), 10 μl of capillary blood was drawn from the earlobe at rest, 3 min after the end of incremental exercise (visit 1) and 2 min after the end of SA and DA (visits 1 and 2).

The rating of perceived exertion (RPE) scores was estimated by the participants using the 15-grade (from 6 to 20) Borg RPE Scale (Borg, 1970; Shephard et al., 1992) 1 min after the end of the incremental exercise (visit 1) and the end of SA and DA (visits 1 and 2). Two minutes after the end of SA and DA (visits 1 and 2), the participants also estimated their subjective feeling (feeling of pleasure or displeasure) about the exercise performed using a Feeling Scale (FS) (Hardy and Rejeski, 1989) consisting of 11 grades between −5 and +5, combined with verbal information (ranging from “Very bad” for −5 to “Very good” for +5).

### Statistical Analysis

Since we test the same group in different conditions and for different variables, we performed a Friedman ANOVA. Then, we used a Wilcoxon matched-pair signed-rank test for intragroup comparisons (condition 1 vs. condition 2). *P-*values < 0.0125 were considered significant since we used a Bonferroni correction. Analyses were performed with Statistica 6.0 software.

## Results

Table 1 shows data at maximal exercise obtained during the incremental test and used to determine P20, P30, P40, and P50.

**TABLE 1:** Measurements taken during the incremental test (visit 1) and used for the dynamic apneas (visit 2).

VO_2_peak (ml.min^–1^.kg^–1^)	VEpeak (l.min^–1^)	HRmax (bpm)	[La] (mmol.l^–1^)	RPE	PVO_2_peak (W)	P20 (W)	P30 (W)	P40 (W)	P50 (W)
47.5 ± 8.2	128.6 ± 28.2	189 ± 7.7	14.9 ± 2.9	16 ± 1.7	232 ± 29.7	46 ± 6.1	70 ± 9.1	93 ± 11.8	116 ± 14.9

*Peak oxygen uptake (VO_2_peak), maximal ventilation (VEpeak), maximal heart rate (HRmax), and blood lactate concentration ([La]) 3 min after the end of the exercise, rating of perceived exertion (RPE), power output achieved at peak oxygen uptake (PVO_2_peak), and power corresponding to 20, 30, 40, and 50% of PVO_2_peak (P20, P30, P40, and P50, respectively).*

**FIGURE 2:**
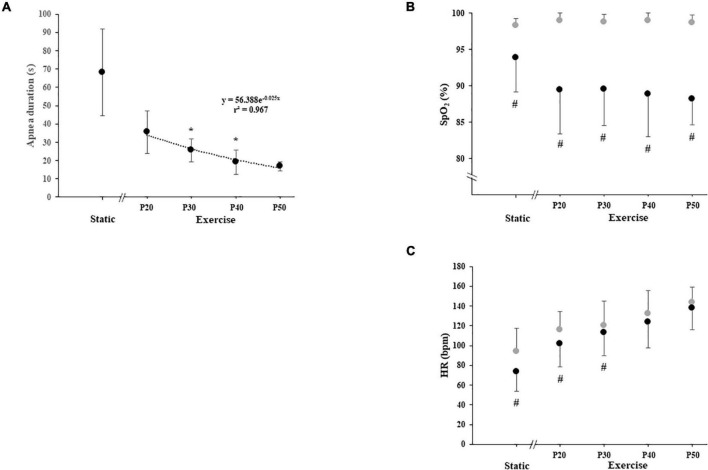
**(A)** Duration of static and dynamic apnea, **(B)** arterial oxygen saturation (SpO_2_), and **(C)** heart rate (HR) with apnea (black circle) and without apnea (gray circle) during static and exercise. The exercise was carried out at 20, 30, 40, and 50% of power output achieved at peak oxygen uptake (P20, P30, P40, and P50, respectively). Significant difference with the apnea duration of the previous power output: *P < 0.0125 (for panel **A**). Significant difference between with and without apnea: #*P* < 0.0125 (for panels **B** and **C**).

Apnea duration was higher during SA (68.1 ± 23.6 s) than during DA, and apnea duration at P20 (35.6 ± 11.7 s) was higher than that at P30 (25.6 ± 6.3 s), P40 (19.2 ± 6.7 s), and P50 (16.9 ± 2.5 s). Apnea duration at P30 was higher than that at P40, and no difference was noted between P40 and P50. The relationship between apnea duration and exercise intensity followed an exponential function (*y* = 56.388e^–0.025^*^x^*; Figure 2).

Static apnea induces a decrease in HR (−22%; *P* = 0.0051). DA performed at P20 and P30 induced a decrease in HR compared with exercise without apnea (−13%, *P* = 0.0081; −6%, *P* = 0.007, respectively). We did not find any apnea effect on HR when dynamic exercises were performed at P40 and P50 (Figure 2).

Static apnea and DA performed at P20 did not modify [La] (*P* = 0.26 and *P* = 0.063, respectively). We found an increase of [La] when we compared rest with P30 values (+97%; *P* = 0.0077) and rest with P40 values (+165%; *P* = 0.0051); but, since we have some missing values (three missing values), despite a very high increase of [La] recorded in seven participants (+247%), we did not find a difference between rest and P50 (*P* = 0.018) (Table 2).

**TABLE 2:** Rating of perceived exertion (RPE), feeling scale (FS), respectively, 1 and 2 min after the end of static apnea (SA) and dynamic apnea (DA) carried out at 20, 30, 40, and 50% of power output achieved at peak oxygen uptake (P20, P30, P40, and P50, respectively), blood lactate ([La]) at rest, 2 min after the end of SA and DA carried out at P20, P30, P40, and P50.

	Rest SA	SA	Rest DA	P20	P30	P40	P50
RPE	/	12.6 ± 3.5	/	11.0 ± 2.2	11.5 ± 2.7	13.6 ± 2.6	13.3 ± 1.9
FS	/	1.1 ± 2.7	/	1.7 ± 1.9	1.2 ± 1.7	0.1 ± 1.9	0.4 ± 2.4
[La] (mmol.l^–1^)	0.96 ± 0.3	0.94 ± 0.3	0.92 ± 0.4	1.20 ± 0.5	1.82 ± 0.5*	2.44 ± 0.8*	3.20 ± 0.7

Significant difference between rest and apnea conditions: *P < 0.0125.

Static apnea induces a decrease in SpO_2_ (−4.4%; *P* = 0.0117). DA induces a decrease in SpO_2_ compared with exercise without apnea at P20 (−9.6%, *P* = 0.0051), P30 (−9.5%; *P* = 0.0051), P40 (−10%; *P* = 0.0070), and P50 (−10.5%; *P* = 0.0051). The SpO_2_ decreases observed during the different DA conditions were always higher compared with SA but were not dependent on the level of exercise intensity (Figure 2). We did not find any effect of experimental conditions on RPE and FS (Table 2).

## Discussion

To the best of our knowledge, this is the first study comparing different exercise intensities during DAs, relationship between duration and exercise intensity, and the associated metabolic parameters. The main result is that the relationship between apnea duration and exercise intensity followed an exponential function. Our outcomes suggest that the best compromise between apnea duration and exercise intensity is around 30% of VO_2_peak exercise in order to induce a sufficient stimulus to lead to hypoxia and [La] increase which can be used during aerobic training.

Stopping ventilation has been shown to initiate the diving response at rest as during exercise (Lin et al., 1983; Lindholm et al., 1999; Lindholm and Lundgren, 2009). Apnea alone is sufficient to trigger the diving response, and it could be modulated by immersion, water temperature, hypoxia, physical activity, and training (Stromme et al., 1970; Schuitema and Holm, 1988; Foster and Sheel, 2005; Andersson and Evaggelidis, 2009; Joulia et al., 2009; Lindholm and Lundgren, 2009). The mechanisms involved are more intense during dynamic than during static conditions (Butler and Woakes, 1987), justifying our interest to compare SA and DA modalities.

The low durations of maximal SA and DA observed during our study confirmed that our participants did not regularly practice apnea. The duration of apnea is determined by the interactions of mechanical, chemical, and psychological factors (Hentsch and Ulmer, 1984; Schagatay, 2009). The longest apneas occur when the most diving response is pronounced (Schagatay and Andersson, 1998). Thus, elite free divers are known to maintain long apnea and to exhibit an accentuated diving reflex (Joulia et al., 2009; Rodriguez-Zamora et al., 2018). The diving response has been shown partly genetically defined (Baranova et al., 2017; Ilardo et al., 2018), but an accentuated diving response can be obtained after a DA training at low exercise intensity in apnea naïve athletes (Joulia et al., 2003). It is very difficult to maintain DA for non-apnea trained (NAT) participants, and if we want to develop apnea as a new modality of training as previously suggested (Lemaître et al., 2010), we need to adapt apnea training to NAT participants. Then, it is necessary to determine the best compromise between apnea duration and the intensity of exercise to limit the duration of the struggle phase of apnea. Later, we observed a significant decrease in the DA durations compared with SA when the intensity of exercise increased (P20: −48%, P30: −60%, and P40: −70%).

Lactatemia was not modified by SA nor by P20. It has been previously shown that SA and DA could induce an increase of lactatemia in trained and NAT participants (Joulia et al., 2003). However, to obtain this increase, the durations of SA and DA needed to induce sufficient hypoxia, and these two conditions were not present in SA and P20 conditions. The diving reflex has been described as accentuated in free divers compared with NAT subjects. Since the diving reflex is an oxygen preservation mechanism, during apneas, free divers are presenting lower O_2_ uptake and CO_2_ production compared with NAT (Joulia et al., 2003). Consequently, the short duration of apneas observed in this study can be partly explained by the higher oxygen uptake and CO_2_ production in our participants and their difficulties to maintain the struggle phase (Hentsch and Ulmer, 1984). Despite very short apnea durations in this study, we observed increases of [La] at P30 (+97%), P40 (+165%), and P50 (+247%) even though for this last condition some missing values did not permit to have significant results. Then, P30 intensity of exercise seems sufficient to stimulate anaerobic glycolysis, justifying the difficulty for participants to maintain apnea for a longer period.

During apnea despite stopping the ventilation, the oxygen uptake was likely unchanged. Then, an SpO_2_ decrease was observed during SA depending on the duration or during DA depending on the duration and the intensity of the exercise (Fitz-Clarke, 2018). In this study, despite a decrease in SpO_2_ during each DA condition, there is no effect of the intensity exercise on SpO_2_. It suggests that the durations are too short to induce a different SpO_2_ decrease. We also know that SpO_2_ is reduced differently depending on the level of apnea practice (Joulia et al., 2015). This study has shown that in order to maximize the apnea stimulus in untrained participants, we should exercise with maximum SA or DA durations. Then, the performed DA training at very low intensity is interesting for NAT participants compared with SA because it is permitting to obtain a greater desaturation suggesting a higher hypoxic stimulus.

Diving bradycardia was the first component of the diving reflex studied (Bert, 1870). It is a primordial oxygen-conserving reflex since less the myocardial tissue oxygen uptake is more the apnea duration will be important (Hoiland et al., 2017). In agreement with previous studies, the greatest bradycardia is observed during SA (−22% vs. rest). This decrease was lower compared with elite free divers since the maximal durations are too short to induce the full development of the bradycardia (Jung and Stolle, 1981), and the bradycardia is known to be more pronounced in elite free divers (Baković et al., 2003; Joulia et al., 2013; Bain et al., 2018). Bradycardia during DA conditions is less marked compared with SA since there is an antagonist effect of exercise on apnea response (Joulia et al., 2009; Alboni et al., 2011). The decrease is also noticeable when comparing DA with the previous exercise performed at the same intensity in normal breathing and because of the effect of exercise and apnea duration on the bradycardias are more marked at P20 (−13%) than at P30 (−6%) confirming Jung and Stolle observations suggesting a minimum of 30 s in apnea to obtain the full development of the diving response (Jung and Stolle, 1981). Likewise, bradycardias were not observed from P40 and P50 since the diving response is limited due to the short apnea time and the intensity of the exercise high enough to counterbalance the effects of apnea in NAT participants.

It has been noted that RPE scores positively correlate with the total immersion time and inversely correlate with the minimum and average HR (Rodríguez-Zamora et al., 2014). Then, we would expect to observe a modified RPE depending on the apnea conditions. Indeed, apnea involves stopping a vital function and constitutes a real “effort” in that psycho-physical stress appears, especially as it is paired with intense exercises (Rodríguez-Zamora et al., 2014). We expect that the stress induced by the apnea associated with the higher intensity of exercise would modify the part of the struggle phase in the apnea, but probably due to the too short duration of the apneas, participants did not feel unpleasant sensations. Since the participants were not apnea trained, the exercise effect is dominant compared with the apnea effect, and participants stopped the apnea at the end of the easy-going phase since unpleasant sensations appear (Hentsch and Ulmer, 1984). FS is poorly used in training sessions. However, an exercise can be felt as hard without being unpleasant, which justifies the addition of an assessment of feeling experienced using an FS. FS values remained unchanged whatever the apnea conditions might be and remained neutral or even had positive values (between “neutral” and “slightly good to good”). The SA and DA were not considered unpleasant. The latter would thus make it possible to carry out an exercise in “bearable” conditions while having an effective stimulation. A strong stimulation of anaerobic glycolysis could take place while limiting the unpleasant effect, often felt during fractional work (HITT) (Saanijoki et al., 2015). Moreover, some non-free-diver athletes also use apnea at the end of training (carried out without necessarily having included apnea in the body of the session) to relax and recover during the so-called calm down phase. It is well known that in free diving training, breathing techniques are derived from pranayama yoga. Since yoga respiration training induces long-lasting modifications of the ventilatory pattern (Villien et al., 2005), we can consider that learning to control the breathing of an individual is also a way of relaxing.

The main limitation of this study is the short durations of the apneas performed by the participants and their number. These short durations are the consequence of the non-apnea practice of the participants, but if a DA training wants to be performed by non-apnea participants, it was necessary to test the different DA conditions in NAT participants.

This study showed that the best compromise between maximal apnea and exercise intensity is around 30% of VO_2_peak exercise in order to induce the higher stimulus for aerobic training. The use of the apnea modality shows that despite the low-intensity exercise and the short duration, we can obtain measurable metabolic modifications. It could be used in sport activities where fast modification of blood flow distributions can appear as during the transitions in triathlon or during isometric phases as downhill mountain bike causing a hypoxia or an ischemia in some territories. Since a low-intensity training associated with apnea induces a metabolic response, it could be interesting to propose this kind of training in rehabilitation after an injury. Moreover, apnea could be an easy way to induce hypoxic preconditioning, which is known to increase the endurance capacity (Wang et al., 2019). Finally, in the field of physical activity conditioning, this modality of exercise would both potentiate the effects of exercise while optimizing the time spent and vary the exercise (which can also be experienced as a challenge). Faster progression is a major source of motivation for patients (Kraemer et al., 2002).

## Data Availability Statement

The raw data supporting the conclusions of this article will be made available by the authors, without undue reservation.

## Ethics Statement

The studies involving human participants were reviewed and approved by the Local Ethics Committee. The patients/participants provided their written informed consent to participate in this study.

## Author Contributions

AG, FP, and FJ conceived and designed the project. KH, AG, and FL performed the data collection. KH, AG, FP, and FJ performed the data analysis and the interpretation of data. All authors contributed to the preparation, critically revised the manuscript, and approved the submitted version.

## Conflict of Interest

The authors declare that the research was conducted in the absence of any commercial or financial relationships that could be construed as a potential conflict of interest.

## Publisher’s Note

All claims expressed in this article are solely those of the authors and do not necessarily represent those of their affiliated organizations, or those of the publisher, the editors and the reviewers. Any product that may be evaluated in this article, or claim that may be made by its manufacturer, is not guaranteed or endorsed by the publisher.

## Abbreviations

DA: dynamic apnea
FS: feeling scale
HR: heart rate
[La]: blood lactate concentration
NAT: non-apnea trained
P20: 20% of the power output achieved at peak oxygen uptake
P30: 30% of the power output achieved at peak oxygen uptake
P40: 40% of the power output achieved at peak oxygen uptake
P50: 50% of the power output achieved at peak oxygen uptake
PVO_2_peak: power output achieved at peak oxygen uptake
RPE: rating of perceived exertion
SA: static apnea
SpO_2_: arterial oxygen saturation
SpO_2_min: minimal value of arterial oxygen saturation
VO_2_peak: peak oxygen uptake.
